# Histone lysine-specific demethylase 1 regulates the proliferation of hemocytes in the oyster *Crassostrea gigas*

**DOI:** 10.3389/fimmu.2022.1088149

**Published:** 2022-12-15

**Authors:** Xiaoyu Gu, Xue Qiao, Simiao Yu, Xiaorui Song, Lingling Wang, Linsheng Song

**Affiliations:** ^1^ Liaoning Key Laboratory of Marine Animal Immunology, Dalian Ocean University, Dalian, China; ^2^ Liaoning Key Laboratory of Marine Animal Immunology and Disease Control, Dalian Ocean University, Dalian, China; ^3^ Dalian Key Laboratory of Aquatic Animal Disease Prevention and Control, Dalian Ocean University, Dalian, China; ^4^ Southern Laboratory of Ocean Science and Engineering (Guangdong, Zhuhai), Zhuhai, China

**Keywords:** *Crassostrea gigas*, hemocytes, LSD1, histone demethylation, cell proliferation

## Abstract

**Background:**

Lysine-specific demethylase 1 (LSD1) is an essential epigenetic regulator of hematopoietic differentiation, which can specifically mono-methylate H3K4 (H3K4me1) and di-methylate H3K4 (H3K4me2) as a transcriptional corepressor. Previous reports have been suggested that it participated in hematopoiesis and embryonic development process. Here, a conserved LSD1 (*Cg*LSD1) with a SWIRM domain and an amino oxidase (AO) domain was identified from the Pacific oyster *Crassostrea gigas*.

**Methods:**

We conducted a comprehensive analysis by various means to verify the function of *Cg*LSD1 in hematopoietic process, including quantitative real-time PCR (qRT-PCR) analysis, western blot analysis, immunofluorescence assay, RNA interference (RNAi) and flow cytometry.

**Results:**

The qRT-PCR analysis revealed that the transcripts of *Cg*LSD1 were widely expressed in oyster tissues with the highest level in the mantle. And the transcripts of *Cg*LSD1 were ubiquitously expressed during larval development with the highest expression level at the early D-veliger larvae stage. In hemocytes after *Vibrio splendidus* stimulation, the transcripts of *Cg*LSD1 were significantly downregulated at 3, 6, 24, and 48 h with the lowest level at 3 h compared to that in the Seawater group (SW group). Immunocytochemical analysis showed that *Cg*LSD1 was mainly distributed in the nucleus of hemocytes. After the *Cg*LSD1 was knocked down by RNAi, the H3K4me1 and H3K4me2 methylation level significantly increased in hemocyte protein. Besides, the percentage of hemocytes with EdU-positive signals in the total circulating hemocytes significantly increased after *V. splendidus* stimulation. After RNAi of *Cg*LSD1, the expression of potential granulocyte markers *Cg*SOX11 and *Cg*AATase as well as oyster cytokine-like factor *Cg*Astakine were increased significantly in mRNA level, while the transcripts of potential agranulocyte marker *Cg*CD9 was decreased significantly after *V. splendidus * stimulation.

**Conclusion:**

The above results demonstrated that *Cg*LSD1 was a conserved member of lysine demethylate enzymes that regulate hemocyte proliferation during the hematopoietic process.

## 1 Introduction

As one of the epigenetic modifications, histone lysine methylation is tightly regulated by methyltransferases and demethylases to remove the methyl group from methylated lysine residues in histone proteins (1, 2). Six lysine residues in histone proteins are linked to chromatin and transcriptional regulation as well as DNA damage response, namely, H3K4, H3K9, H3K27, H3K36, H3K79, and H4K20 (1). Histone lysine methylation determines the opening states of specific genome regions, which regulate gene activation and repression events in cell cycle, genome stability, nuclear architecture, and hematopoiesis (2). In vertebrates, this modification is dynamically regulated by a series of enzymes, such as poly-comb repressive complex 2 (PRC2), disruptor of telomere silencing 1-like (Dot1l), and mixed-lineage leukemia 1 (MLL1), which are essential in regulating hematopoietic stem cell (HSC) activity and keeping them in a quiescent state (3). Histone methylation has been considered as an enzymatically reversible process since the discovery of lysine-specific demethylate 1 (LSD1), providing compelling evidence that this modification was dynamically regulated (4).

LSD1 has been widely found in many vertebrates and invertebrates, which shows a highly conserved structure in the organisms from yeast to human (5–7). Previous reports have already demonstrated that LSD1 was originally considered to demethylate dimethyl lysine 4 of histone H3 as a component of the corepressor element silencing factor (CoREST) corepressor complex (8). In addition to its ability to demethylate H3K4me1 and H3K4me2, it has the ability to specifically catalyze the demethylation of H3K9me1 and H3K9me2 in different contexts (9, 10). The structure of the vertebrate LSD1 protein contains three major domains, namely, an N-terminal SWIRM domain, a C-terminal amine oxidase-like (AOL) domain, and a central protruding Tower domain (11). The N-terminal SWIRM (Swi3p, Rsc8p, and Moira) domain is important for the protein stability and the interaction with other proteins (11). Recent studies revealed that the AOL domain is functionally divided into two subdomains, the FAD binding domain and the substrate binding domain (12). Together, the two subdomains form a large cavity at their interface and create a catalytic center (13). The Tower domain is a protruding structure that is inserted into the AOL domain of LSD1 and represents an essential surface for the binding of the LSD1 partner protein, such as CoREST (11, 13). As a component of multiple transcriptional regulatory complexes, LSD1 is involved in transcriptional regulation (14). Lysine-specific demethylate 2 (LSD2) is another homolog protein of LSD1, which is also a FAD-dependent amino oxidase demethylase with the strict ability of demethylating H3K4me1 and H3K4me2 (15). Different from LSD1, the amine oxidase domain of LSD2 lacks the protruding Tower structure in the C-terminal but contains a zinc finger domain (Zn-CW) in the N-terminal, which does not exist in LSD1 (15, 16). LSD2 is involved in other regulatory processes. For example, LSD1 generally acts as a transcriptional activator and a repressor at the specific region of the gene promoter or enhancer, whereas LSD2 is preferentially located in the transcriptionally activated region of genes (16). In invertebrates, some homologue genes of LSD1 have been identified from *Drosophila melanogaster* (*D. melanogaster*) and *Caenorhabditis elegans* (*C. elegans*) with similar structural features to those from vertebrates (5, 17).

Accumulating reports have supported the idea that LSD1 plays a critical role in cell proliferation and differentiation by associating with multiple factors that are correlated with particular chromatin environments (14, 18, 19). For instance, one of the best-characterized transcription repressor complexes is the CoREST, which is necessary for cell-lineage determination and differentiation during pituitary organogenesis (20). The LSD1–CoREST complex is also essential in nucleosome demethylation by contacting both histone and DNA (21, 22). The LSD1 biological function was exercised by cooperating with different proteins. For example, the hematopoietic related regulator Sal-like Protein 4 (SALL4) dynamically recruits LSD1 to negatively regulate its specific target genes (23). Moreover, stem cell leukemia (SCL, also known as TAL1) dynamically interacted with LSD1 to regulate hematopoietic transcription and differentiation programs during hematopoiesis and leukemogenesis (24). Furthermore, during erythroid differentiation, LSD1 demethylates the 1S promoter region of the transcription factor GATA-2 to suppress the expression of GATA-2 (25). It was also reported that LSD1 regulates the balance between self-renewal and differentiation in human ESCs by regulating some developmental genes at the regulatory regions of H3K4 and H3K27 (26). In mouse bone marrow, the deficiency of LSD1 was found to enhance progenitor cells’ proliferative behavior that led to an increase in the number of hematopoietic stem and progenitor cells (HSPCs) (27). In addition, it was reported that the ESCs lacking LSD1 failed to differentiate into embryoid bodies, and the loss of LSD1 led to embryonic lethality (20, 28). In invertebrates, most of the investigations of LSD1 homologs are focused on embryo development (5, 29). For example, the LSD1 mutants of *Drosophila* were found to be sterile and their ovary development was severely impaired (30). During the development of *Drosophila* follicle cells, the differentiation of follicle cell progenitors is suppressed by LSD1 and CoREST to maintain the progenitor state until they have completed an appropriate number of divisions (31). It is speculated that the regulation of LSD1 on progenitor cell proliferation is conserved in vertebrates and invertebrates. Nonetheless, research about the involvement of LSD1 in invertebrate hematopoiesis processes is still very limited.

The pacific oyster *Crassostrea gigas* (*C. gigas*), as an important economic marine bivalve species, has evolved an effective immune system to defend the invasion of pathogens (32). Oyster hemocytes are considered as the main immune cells that are primarily responsible for defense against pathogens (33). The replenishment of hemocytes from hematopoiesis is very important for maintaining immune homeostasis (34, 35). In the present study, an LSD1 homologue (*Cg*LSD1) was identified from the Pacific oyster *C. gigas* with the following main objectives : (1) to characterize its sequence structure and the expression pattern in tissues of adult oyster and embryotic development , (2) to determine the demethylase activity of *Cg*LSD1, and (3) to clarify the involvement of *Cg*LSD1 in hemocyte proliferation and provide more lines of evidence to further understand the regulation of hematopoietic process in molluscs.

## 2 Materials and methods

### 2.1 Animals, tissues, and stimulation collection

All experiments were performed in accordance with the approval and guidelines of the Ethics Review Committee of Dalian Ocean University. The adult Pacific oysters were collected from the local aquaculture farm (Dalian, Liaoning province, China). The SPF female Kunming mice (6 weeks old) were obtained from Dalian Institute of Drug Control. According to the lab’s previous report (36), these animals were adapted appropriately for 1 week before experiments.

The immune stimulation experiment was conducted according to the previous report (37). Briefly, 140 oysters were randomly divided into two groups (the *V. splendidus* group and the SW group), each receiving an individual injection of 100 μl of live *V. splendidus* suspension (2 × 10^6^ CFU/ml in sterile seawater) and 100 μl of sterile seawater, respectively. The total hemocytes were collected at 0, 3, 6, 12, 24, 48, and 72 h post-injection for RNA extraction. Every experiment group contained nine oysters that were randomly divided into three replicants, and each replicant contained three oysters randomly. The hemocyte samples were collected from three oysters and pooled together as one replicant. Six tissues, namely, gonad, adductor muscle, gills, digestive gland, hemocytes, and mantle, were collected from untreated adult oysters. Embryo and larvae samples in different stages during the embryonic development (zygote, 0 hpf; four-cell, 1 hpf; eight-cell, 2 hpf; blastula, 4 hpf; gastrula, 8 hpf; trochophore 1, 12 hpf; trochophore 2, 16 hpf; early D-veliger larvae, 24 hpf) were collected based on previous reports (38).

### 2.2 RNA isolation and cDNA synthesis

According to our previous report (39), the total RNA was extracted from six adult tissues, embryo, and larvae with the TRIzol™ Reagent (TransGen, China) and synthesized into cDNA with TransScript One-Step gDNA Removal and the cDNA Synthesis Super Mix Kit (TransGen, China). The integrities and concentration of the RNA samples were estimated by a Nanodrop 2000 Spectrophotometer (ThermoFisher, USA). The obtained cDNA template was stored at −80°C for the subsequent experiment.

### 2.3 Sequence analysis of *Cg*LSD1

The predicted lysine-specific histone demethylase 1A (LOC105346267) was identified from the *Crassostrea gigas* genome database of the National Center for Biotechnology Information (https://www.ncbi.nlm.nih.gov/). The deduced amino acid sequences were analyzed with the Expert Protein Analysis System (ExPASY, http://www.expasy.org/), and the structure domains of the LSD1 protein were predicted by SMART version 5.1 (http://smart.embl-heidelberg.de/). In addition, the presumed tertiary structure of *Cg*LSD1 was established by the SWISS-MODEL prediction algorithm (http://swissmodel.expasy.org/). The homology of the LSD1 family was conducted by the BLAST algorithm (http://www.ncbi.nlm.gov/blast). Multiple alignment of LSD1s was conducted by the ClustalW Multiple Alignment program (http://www.ebi.ac.uk/clustalw/). A Neighbor-Joining (NJ) phylogenetic tree was constructed by the MEGA 6.0 software (40).

### 2.4 Quantitative real-time PCR analysis

The qRT-PCR was performed to detect the mRNA transcripts of *Cg*LSD1. In our previous studies, *Cg*AATase (XM_011424773.3) was found to be a potential marker of granulocytes (41). *Cg*SOX11 (XM_011446901.3) was another specific marker for granulocytes that is located in the nucleus of granulocytes (unpublished data). *Cg*CD9 (XM_011432003.3) was identified as a specific surface marker for agranulocytes (42). The cytokine-like factor *Cg*Astakine (JH818724.1) was considered a cytokine, which accelerates hemocyte proliferation (43). The corresponding primers are shown in **Table 1**. A fragment of *Cg*EF (NP_001292242.2) with corresponding primers P5 and P6 (**Table 1**) was used as an internal reference (44). The mRNA expression level of each gene was calculated by the 2^−ΔΔCT^ method according to a previous report (45).

**Table 1:** Sequences of the primers used in this study.

	Primer	Sequence (5′-3′)
P1	*Cg*LSD1-SWIRM-F	AAACCTCAACCTACCCCTC
P2	*Cg*LSD1-SWIRM-R	GGCTCCGATTATTATCACT
P3	re*Cg*LSD1-SWIRM-F	CGCGGATCCAAACCTCAACCTACCCCTC
P4	re*Cg*LSD1-SWIRM-R	CCGCTCGAGGGCTCCGATTATTATCACT
P5	EF-RT-F	AGTCACCAAGGCTGCACAGAAAG
P6	EF-RT-R	TCCGACGTATTTCTTTGCGATGT
P7	*Cg*RT-LSD1-F	TCCACCTCAGTCCCAAAAAGTC
P8	*Cg*RT-LSD1-R	GTTGATGTAGCCAAACCTCTCTAAGTA
P9	*Cg*CD9-RT-F	CACAAAGTATTCCGATGCCGA
P10	*Cg*CD9-RT-R	CAGATTCCAGCCCCAAGTAAGA
P11	*Cg*AATase-RT-F	CAACGACTGTCTCAAGATGCGG
P12	*Cg*AATase-RT-R	ACAAACCATCGCCTCCGTCA
P13	*Cg*SOX11-RT-F	CTGGGTAAACGCTGGAAAACG
P14	*Cg*SOX11-RT-R	TCTGCTTCATACGGTCGGTGC
P15	*Cg*Astakine-RT-F	GACACGAGTTGCCCCACC
P16	*Cg*Astakine-RT-R	GCTACCGTCGAACAGGATT
P17	T7-LSD1-F	TAATACGACTCACTATAGGGATCATGC GTACTTAGAGAGGTTTG
P18	T7-LSD1-R	TAATACGACTCACTATAGGGATCACGCT GCATTCAGATCTCT
P19	T7-EGFP-F	TAATACGACTCACTATAGGGATCCGACG TAAACGGCCACAAGT
P20	T7-EGFP-R	TAATACGACTCACTATAGGGATCCTTGTA CAGCTCGTCCATGC

### 2.5 Expression and purification of the recombinant protein

The full length of *Cg*LSD1 was obtained with the specific primers P1 and P2 (**Table 1**). Another pair of specific primers P3 and P4 (**Table 1**) with *Bam*HI and *Xho*I cleavage sites was designed to amplify the cDNA of the SWIRM domain of *Cg*LSD1 and cloned into pET-30a expression vector. *E. coli* Transetta (DE3) (TransGen Biotech, China) was used to express the recombinant protein of *Cg*LSD1-SWIRM (designated as r*Cg*LSD1-SWIRM). After isopropyl-β-D-thiogalactoside (IPTG) induction, the r*Cg*LSD1-SWIRM protein was purified by Ni^+^ affinity chromatography (Sangon, China). The concentration of r*Cg*LSD1-SWIRM protein was verified by the BCA method (Beyotime, China) and stored at −80°C for the preparation of antibody.

### 2.6 The preparation of *Cg*LSD1 antibody and Western blot analysis

Kunming mice were immunized four times with r*Cg*LSD1-SWIRM protein to acquire the polyclonal antibody of *Cg*LSD1 according to a previous description (46). After four injections of r*Cg*LSD1-SWIRM, blood was quickly taken from the mice and tilted placed at 4°C for 7 h. The serum was harvested by centrifugation at 4°C, 3,000 × *g* for 30 min and the anti-*Cg*LSD1 serum was stored at −80°C.

The specificity of *Cg*LSD1 polyclonal antibody was verified by Western blot assay according to a previous report (47). The total hemocyte proteins were separated by SDS-PAGE and transferred onto a nitrocellulose (NC) membrane. After blocking with 5% skimmed milk, the membrane was incubated with the polyclonal antibody of *Cg*LSD1 at 37°C for 3 h (1:1,000 diluted in 5% skimmed milk). After three washes, the membrane was incubated with the antibody of Goat anti-mouse IgG conjugated with HRP (Beyotime, China) at 37°C for 2 h (1:1,000 diluted in 5% skimmed milk). The protein bands in the image were developed by Super ECL Detection Reagent (Beyotime, China) and captured by the Amersham Imager 600 system (GE Healthcare, USA).

### 2.7 Immunofluorescence assay

According to a previous report (48), immunocytochemistry assay was conducted to observe the subcellular localization of *Cg*LSD1 in hemocytes. To be brief, hemocytes were collected from six oysters and resuspended in modified Alsever’s solution. Then, the resuspension was deposited onto a glass slide and kept at room temperature (37°C) for 2 h to attach the slide. After fixing with 4% paraformaldehyde (Sangon, China) and permeabilizing with 0.5% Triton X-100, the hemocytes were blocked with 3% fetal bovine serum albumin (BSA, Sangon, China) for 2 h. The hemocytes were incubated with the polyclonal antibody of *Cg*LSD1 with a dilution of 1:500 in 3% BSA for 1 h. After three washes, the samples were incubated with secondary antibody Alexa Fluor 488-labeled Goat anti-mouse antibody (Beyotime, China). The nuclei were stained with DAPI (Beyotime, China). Finally, the slides were observed using a fluorescence microscope (Zeiss, Germany).

### 2.8 RNA interference of *Cg*LSD1

The RNA interference experiment was performed as previously described (44). T7 promoter linked primers P17 and P18 (**Table 1**) were used to amplify the DNA fragment of *Cg*LSD1 (714 bp), which were used as a template to synthesize dsRNA of *Cg*LSD1. The specific primers P19 and P20 (**Table 1**) were used to amplify the enhanced green fluorescent protein (EGFP) DNA (657 bp) fragment from the pAcGFP1 vector (Clontech, Japan) as a control. The dsRNAs of *Cg*LSD1 and EGFP were synthesized by the Transcription T7 Kit *in vitro* (Takara, China) according to the manufacturer’s instruction. The final concentration of dsRNAs of *Cg*LSD1 and EGFP was adjusted to 1 μg/μl.

Twenty-seven oysters were randomly divided into three groups (PBS group, dsEGFP group, and dsLSD1 group), which received an injection of 100 μl of PBS, dsRNA (100 μg) of *Cg*LSD1, and EGFP in PBS, respectively. Twelve hours after the first injection, the oysters received another injection with dsRNA of *Cg*LSD1 and EGFP to strengthen the effect of RNAi. After the secondary injection of dsRNA, each oyster received a 100-μl injection of 5-ethynyl-2′-deoxyuridine (EdU, 2 mM, Beyotime, China) at 6 h and a 100-μl injection of *V. splendidus* suspension (resuspended in PBS) at 12 h. Then, the hemocytes were collected for RNA extraction, Western blot analysis,and flow cytometry detection. The commercial H3K4me1 antibody (Rabbit Polyclonal Antibody, Cat#AF5635, Beyotime, China) and H3K4me2 antibody (Rabbit Polyclonal Antibody, Cat#AF5653, Beyotime, China) were employed as the primary antibody to detect methylation level by Western blot assay. β-tubulin (Rabbit Monoclonal Antibody, Cat#AF1216 Beyotime, China) was used as a control.

### 2.9 EdU labeling and cell proliferation detection

According to our previous report (49), the cell population in scatter diagram (P1 in **Figure S1**), the left bottom of the density plots (P2 in **Figure S1**), and the top right corner of the density plots (P4 in **Figure S1**) were considered as the total oyster hemocytes, agranulocytes, and granulocytes, respectively. EdU labeling was conducted according to the protocol of the BeyoClick™ EdU Cell Proliferation Kit with Alexa Fluor 488 (Catalog Number C0071S, Beyotime, China). The positive signals of EdU indicated the newborn hemocytes, which have the ability to proliferate. The EdU-positive signal was examined by a BD FACS Aria flow cytometer (BD Biosciences, America). The PBS group injected with EdU and *V. splendidus* without EdU labeling was used as the negative group. The P1 plot of the histogram (**Figure S1**) was considered as EdU-positive signal hemocytes.

### 2.10 Statistical analysis

All data were given as mean ± S.D. (*N* = 3) and analyzed by SPSS 20. After statistics were analyzed by ANOVA and Tukey *t*-test comparisons, the differences between the experiment group and the control group were accepted (significant at *p* < 0.05 and very significant at *p* < 0.01).

## 3 Results

### 3.1 The sequence and phylogenetic characteristics of *Cg*LSD1

**Figure 1:**
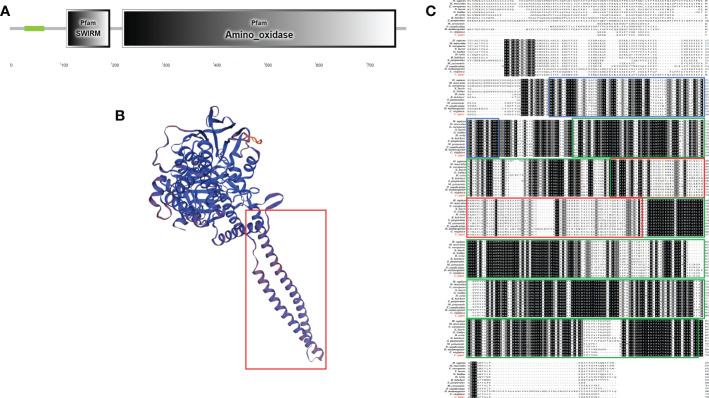
Molecular characteristics of *Cg*LSD1. **(A)** Deduced amino acid sequence domains of *Cg*LSD1 predicted by SMART (http://smart.emblheidelberg.de/). **(B)** The presumed tertiary structure of *Cg*LSD1 established using the SWISS-MODEL prediction algorithm; the predicted LSD1-Tower domain is in the red box. **(C)** Multiple sequence alignment of *Cg*LSD1 from various species (Using the ClustalX 2.0 program). The black shadow region means all sequences share the same amino acid residue; the gray and light-gray shadow indicates the amino acids with a similarity of more than 75% and 50%, respectively. The following are the proteins analyzed: *H. sapiens* (*Homo sapiens*, Accession No. NP_001009999.1); *M. musculus* (*Mus musculus*, Accession No. NP_001334150.1); *E. europaeus* (*Erinaceus europaeus*, Accession No. XP_016046134.1); *G. gallus* (*Gallus*, Accession No. XP_417719.6); *X. laevis* (*Xenopus laevis*, Accession No. XP_018105073.1); *D. rerio* (*Danio rerio*, Accession No. NP_001229924.1); *B. belcheri* (*Branchiostoma belcheri*, Accession No. XP_019628217.1); *S. purpuratus* (*Strongylocentrotus purpuratus*, Accession No. XP_030850350.1); *P. canaliculate* (*Pomacea canaliculate*, Accession No. XP_025088904.1); *C. gigas* (*Crassostrea gigas*, Accession No. XP_011453074.2); *C. virginica* (*Crassostrea virginica*, Accession No. XP_022292745.1); *M. yessoensis* (*Mizuhopecten yessoensis*, Accession No. XP_021359369.1); and *D. melanogaster* (*Drosophila melanogaster*, Accession No. AAM11190.1). The SWIRM domain is in the blue box, the AO domain is in the green box, and the predicted LSD1-Tower domain is in the red box.

The ORF of *Cg*LSD1 was cloned from the oyster *C. gigas* based on the genome information from NCBI (LOC105346267). The full length of *Cg*LSD1 was 2,337 bp, encoding a polypeptide of 778 amino acids with a theoretical relative molecular weight of 87.22 kDa and an isoelectric point of 6.23. The predicted *Cg*LSD1 protein contains one SWIRM domain (amino acids 107–206) and one amine oxidase (AO) domain (amino acids 221–757) (**Figure 1A**). There were 22 α-helixes and 35 β-sheets in the predicted 3D structure of monomer *Cg*LSD1 (**Figure 1B**). *Cg*LSD1 was predicted to form a protruding structure with two α-helices in the AO domain as a Tower domain (amino acids 341–446) (**Figure 1B**). The amino acid sequence of *Cg*LSD1 shared 30.0%–94.0% sequence similarity with those of LSD1s from other organisms, such as 94.99% with *Crassostrea virginica* (*C. virginica*), 78.17% with *Mizuhopecten yessoensis* (*M. yessoensis*), and 69.48% with *Strongylocentrotus purpuratus* (*S. purpuratus*) (**Figure 1C**).

**Figure 2:**
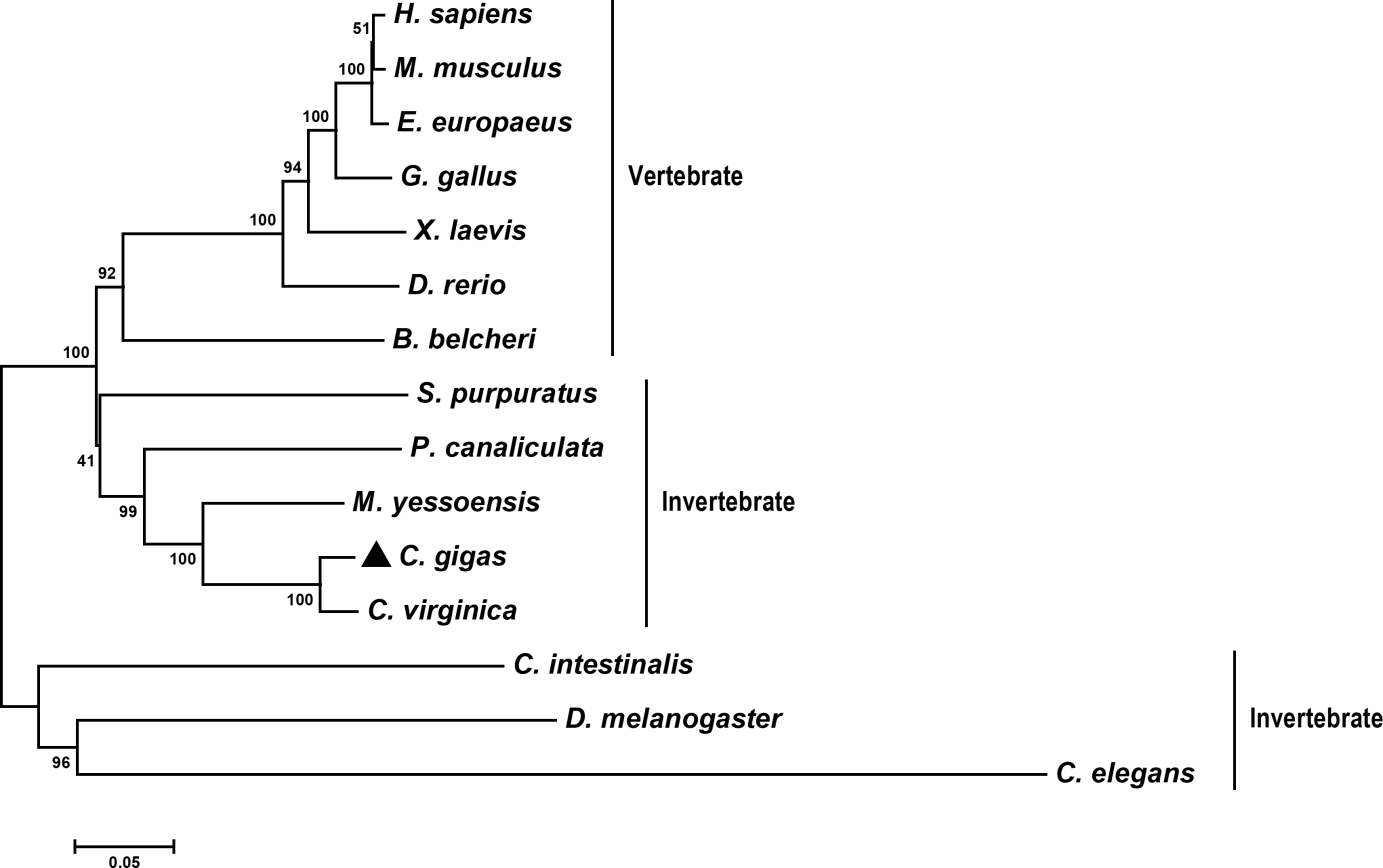
Phylogenetic relationships of *Cg*LSD1 with LSD1s from other species. Other proteins analyzed are listed as follows: *C. elegans* (*Caenorhabditis elegans*, Accession No. NP_493366.1) and *C. intestinalis* (*Ciona intestinalis*, Accession No. XP_002131150.1).

The evolutionary relationship of *Cg*LSD1 and other LSD1s was analyzed by constructing a phylogenetic tree. Fifteen LSD1 sequences from different organisms were selected to construct an NJ polygenetic tree. The result showed that the selected LSD1 members were divided into two branches (vertebrate and invertebrate). *Cg*LSD1 was primarily clustered with the LSD1 homologs from *C. virginica*, *M. yessoensis*, and *Pomacea canaliculat*e (*P. canaliculate*) to form a mollusc cluster in the invertebrate branch (**Figure 2**).

### 3.2 The expression level of *Cg*LSD1 in different tissues and embryonic development stages of *C. gigas*

**Figure 3:**
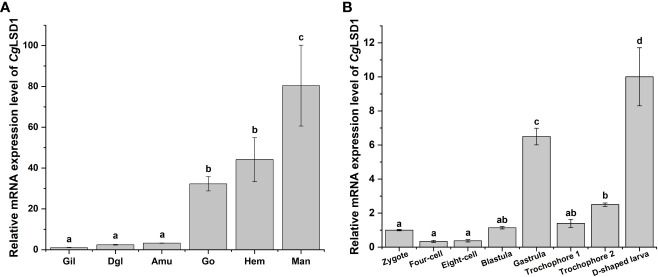
The expression profiles of *Cg*LSD1 mRNA in different tissues. **(A)** The expression profiles of *Cg*LSD1 mRNA in different tissues were examined by qRT-PCR analysis. Gil: gills; Dgl: digestive gland; Amu: adductor muscle; Man: mantle; Hae: hemocytes; Go: gonad. **(B)** The expression pattern of *Cg*LSD1 mRNA in different developmental stages was examined by qRT-PCR analysis. Zygote, 0 hpf; Four-cell stage, 1 hpf; Eight-cell stage, 2 hpf; Blastula, 4 hpf; Gastrula, 8 hpf; Trochophore 1 stage, 12 hpf; Trochophore 2 stage, 16 hpf; D-veliger larvae, 24 hpf. Vertical bars show the mean ± S.D. (*n* = 3). Data with different letters (a, b, c, etc.) indicate significant differences compared with other groups (*p* < 0.05, ANOVA).

qRT-PCR was performed to examine the *Cg*LSD1 mRNA in different tissues of adult oysters. The mRNA transcripts of *Cg*LSD1 were ubiquitously expressed in all tested tissues, including gills, digestive gland, adductor muscle, gonad, hemocytes, and mantle (**Figure 3A**). The highest expression level was detected in the mantle, which was approximately 80.40-fold (*p* < 0.05) of that in gills. In addition, the relative expression level of *Cg*LSD1 in hemocytes and gonad was 44.14-fold (*p* < 0.05) and 32.23-fold (*p* < 0.05) of that in gills, respectively. However, there was no significant difference in *Cg*LSD1 mRNA expression in the digestive gland, adductor muscle, and gills.

The mRNA transcripts of *Cg*LSD1 were also tested during the *C. gigas* embryonic period and larval stages, including zygote, four-cell, eight-cell, blastula, gastrula, trochophore, and D-veliger stages. Compared to the larval stages, the relative mRNA transcripts of *Cg*LSD1 did not change significantly at the zygote, four-cell, eight-cell, and blastula stages (**Figure 3B**). The mRNA expression level of *Cg*LSD1 increased rapidly in the gastrula stage, which was 6.49-fold (*p* < 0.05) of that in the zygote stage. In the larvae stages, the mRNA expression level of *Cg*LSD1 increased significantly at the late trochophore stage, which was 2.49-fold (*p* < 0.05), and reached the highest level in the D-veliger stage, which was 10.00-fold (*p* < 0.05) of that in zygote.

### 3.3 The temporal expression of *Cg*LSD1 in hemocytes after *V. splendidus* stimulation

**Figure 4:**
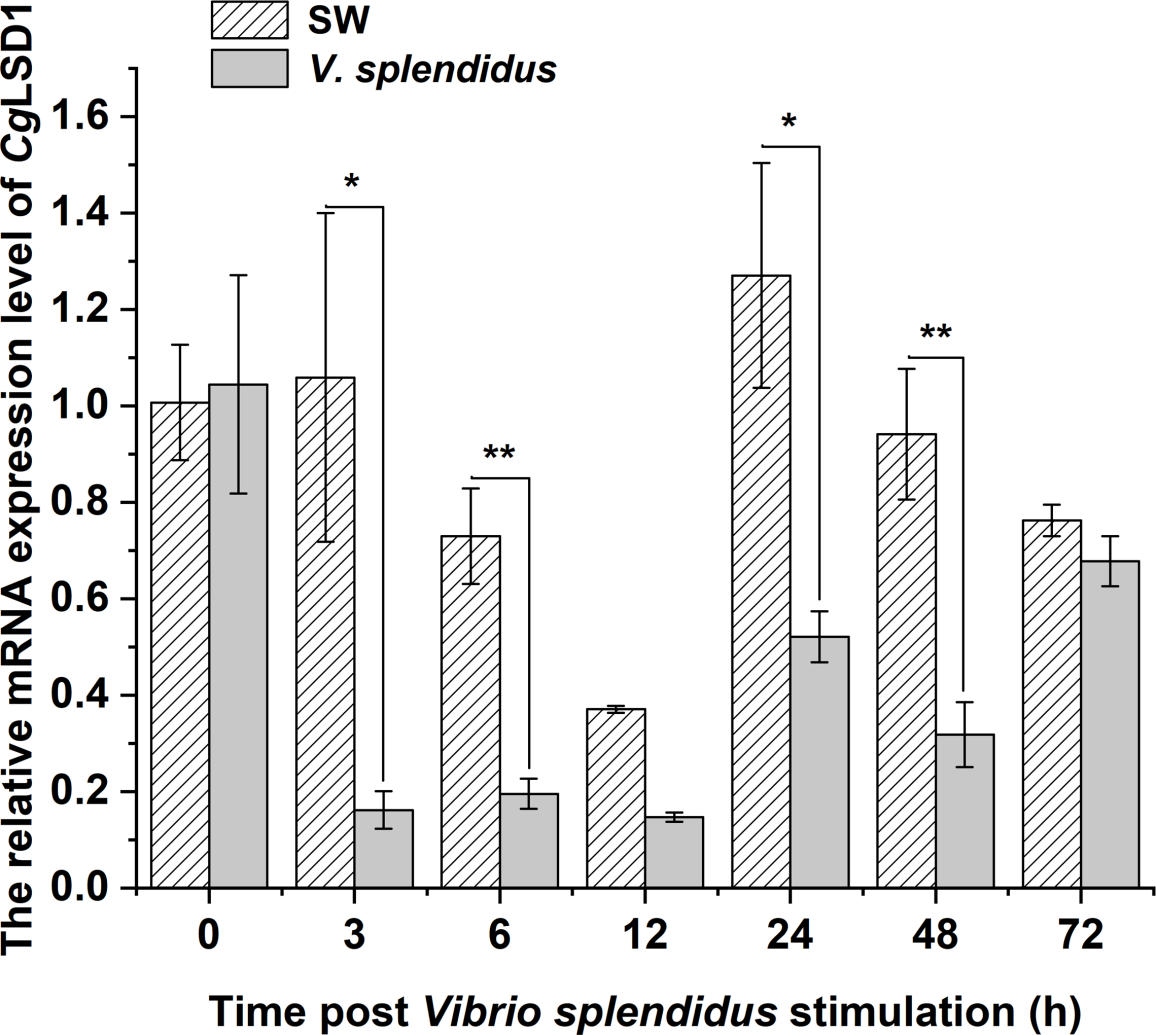
The expression level of *Cg*LSD1 in hemocytes after *V. splendidus* stimulation. Vertical bars show the mean ± S.D. (n = 3); *p < 0.05; **p < 0.01.

In hemocytes, the mRNA transcripts of *Cg*LSD1 were detected at 0, 3, 6, 12, 24, 48, and 72 h after *V. splendidus* stimulation. The mRNA expression level of *Cg*LSD1 was significantly downregulated at 3 and 6 h, which was 0.15-fold (*p* < 0.05) and 0.27-fold (*p* < 0.01) of that in the SW group, respectively. Then, the *Cg*LSD1 transcripts reached the lowest expression level at 12 h although there was no significant difference between the SW group and the *V. splendidus* group (0.56-fold, *p* > 0.05). Then, the mRNA expression level of *Cg*LSD1 increased at 24 h and 48 h but still significantly lower than that in the control group, which was 0.41-fold (*p* < 0.05) and 0.34-fold (*p* < 0.05) of that in the SW group, respectively. At 72 h after *V. splendidus* stimulation, the expression level of *Cg*LSD1 recovered to the normal level with no significant difference compared with that in the SW group (0.89-fold, *p* > 0.05) (**Figure 4**).

### 3.4 The prokaryotic expression and polyclonal antibodies preparation of *Cg*LSD1

**Figure 5:**
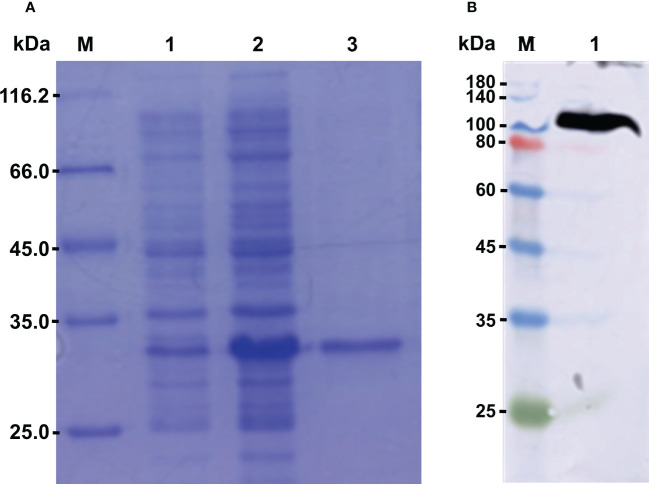
The SDS-PAGE and Western blot analysis of r*Cg*LSD1. **(A)** SDS-PAGE analysis of the *Cg*LSD1-SWIRM recombinant protein. M: protein molecular standard (116.7 kDa); Lane 1: negative control for r*Cg*LSD1-SWIRM (without induction); Lane 2: induced r*Cg*LSD1-SWIRM; Lane 3: purified r*Cg*LSD1-SWIRM. **(B)** Western blot analysis of *Cg*LSD1 antibody. M: protein molecular standard (180.0 kDa); Lane 1: Western blot based on hemocyte protein.

The recombinant protein of *Cg*LSD1-SWIRM (r*Cg*LSD1-SWIRM) was expressed by *E. coli* Transetta (DE3) and purified by the Ni^+^ affinity chromatography. After being analyzed by 15% SDS-PAGE, a distinct band of 34 kDa was observed, which was coincident with the predicted molecular weight of r*Cg*LSD1-SWIRM with 6 × His tag (**Figure 5A**). The preparation of *Cg*LSD1 polyclonal antibody was obtained by the purified r*Cg*LSD1-SWIRM, and the specificity of anti-*Cg*LSD1 antibody was examined by Western blot analysis with hemocyte protein. A distinct band of about 100 kDa was observed, which was consistent with the predicted molecular weight of *Cg*LSD1, indicating the high specificity and efficiency of anti-*Cg*LSD1 polyclonal antibodies (**Figure 5B**).

### 3.5 The subcellular localization of *Cg*LSD1 in oyster hemocytes

**Figure 6:**
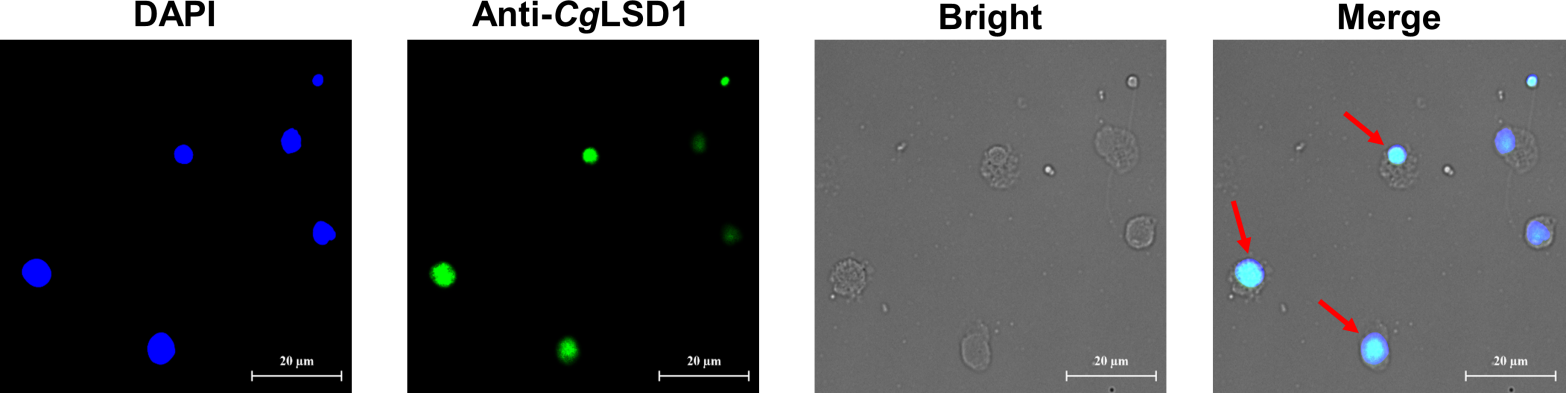
The distribution of *Cg*LSD1 in hemocytes. Immunocytochemistry assay was performed to determine the distribution of *Cg*LSD1 in hemocytes. The positive signals of *Cg*LSD1 were observed in green and the nucleus dyed by DAPI were shown in blue. The right panel shows a magnified image and the red arrows point to the hemocytes with *Cg*LSD1-positive signals.

Immunocytochemistry assay was performed to determine the subcellular localization of the *Cg*LSD1 protein in hemocytes of the oyster *C. gigas*. The *Cg*LSD1 protein labeled with Alexa Fluor 488 is shown in green and the nucleus stained with DAPI is shown in blue. The positive signals of *Cg*LSD1 with green fluorescence were dominantly distributed in the nucleus of hemocytes, while no fluorescence signal of *Cg*LSD1 was observed in the cytoplasm (**Figure 6**).

### 3.6 The alternation of histone methylation expression of H3K4me1 and H3K4me2 after *Cg*LSD1 was knocked down

**Figure 7:**
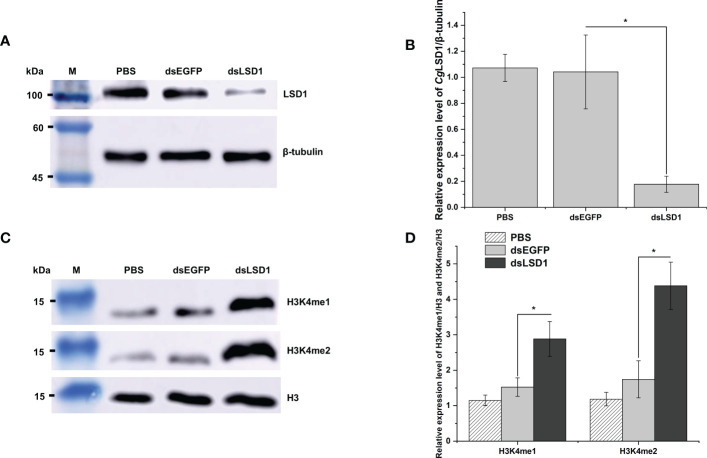
The detection of histone methylation expression in *Cg*LSD1-RNAi oysters in hemocytes. **(A)** Western blot analysis of *Cg*LSD1. β-tubulin was used as control. **(B)** The gray-scale analysis of *Cg*LSD1 and β-tubulin protein level using ImageJ (the ratio of *Cg*LSD1 to β-tubulin). Vertical bars show the mean ± S.D. (*n* = 3); *p < 0.05. **(C)** Western blot analysis of H3K4me1 and H3K4me2; β-tubulin was used as control. **(D)** The gray-scale analysis of H3K4me1, H3K4me2, and H3 protein level using ImageJ (the ratio of H3K4me1 and H3K4me2 to H3). Vertical bars show the mean ± S.D. (*n* = 3); *p < 0.05.

The dsRNA was designed and employed to knock down the expression of *Cg*LSD1. The total protein was extracted from hemocytes for Western blot analysis. The distinct band of *Cg*LSD1 was thinner in the dsLSD1 group than in the dsEGFP group (**Figure 7A**). The relative abundance of *Cg*LSD1/*Cg*β-tubulin decreased significantly, which was 0.17-fold (*p* < 0.05) of that in the dsEGFP group (**Figure 7B**). Meanwhile, the distinct bands of H3K4me1 and H3K4me2 were observed with the molecular weight of 15 kDa, which were much thicker in the dsLSD1 group than in the dsEGFP group (**Figure 7C**). The relative abundance of *Cg*H3K4me1/*Cg*H3 and *Cg*H3K4me2/*Cg*H3 increased significantly, which was 1.89-fold and 2.51-fold (*p* < 0.05) of that in the dsEGFP group, respectively. These results indicated that *Cg*LSD1 was able to mono-demethylate and di-demethylate H3K4 (**Figure 7D**).

### 3.7 The percentage of hemocytes with EdU-positive signals in *Cg*LSD1-RNAi oysters after *V. splendidus* stimulation

**Figure 8:**
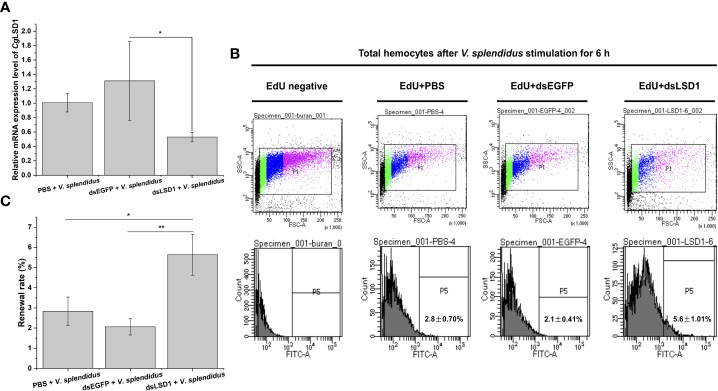
Hemocyte proliferation in *Cg*LSD1-RNAi oysters after *V. splendidus* stimulation. **(A)** The changes of mRNA expression of *Cg*LSD1 in hemocytes after RNAi of *Cg*LSD1 and *V. splendidus* injection. **(B)** New generated hemocytes labeled with EdU were analyzed by flow cytometry. The hemocytes of PBS and *V. splendidus*-injected oysters were used to identify the positive region. **(C)** The statistical analysis of the number of hemocytes labeled with EdU from flow cytometry. Vertical bars show the mean ± S.D. (*n* = 3); *p < 0.05; **p < 0.01.

In order to explore the relationship of *Cg*LSD1 and hematopoiesis, the specific dsRNA targeting *Cg*LSD1 was designed and employed as previously described. The relative mRNA expression level of *Cg*LSD1 in hemocytes was significantly downregulated in *Cg*LSD1-RNAi oysters after *V. splendidus* injection, which was about 0.44-fold of that in the dsEGFP group (*p* < 0.05) (**Figure 8A**). The hemocytes with EdU-positive signals indicate the renewal of circulating hemocytes that were determined by flow cytometry. The percentage of EdU-positive hemocytes in the dsLSD1 group was 5.63%, which was 1.99-fold (*p* < 0.05) and 2.73-fold (*p* < 0.01) of that in the PBS group and dsEGFP group, respectively (**Figures 8B, C**).

### 3.8 The expression of hematopoiesis-related genes in *Cg*LSD1-RNAi oysters after *V. splendidus* stimulation

**Figure 9:**
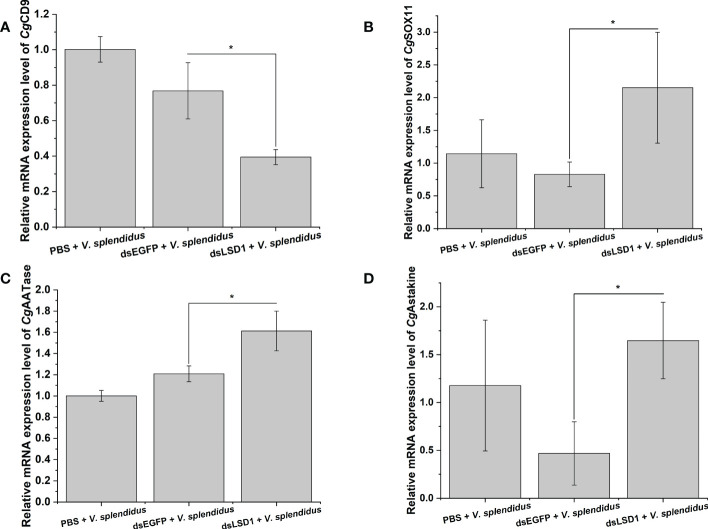
The expression level changes of hemocyte-related gene expression in *Cg*LSD1-RNAi oysters after *V. splendidus* stimulation. **(A)** The mRNA expression level of *Cg*CD9. **(B)** The mRNA expression level of *Cg*SOX11. **(C)** The mRNA expression level of *Cg*AATase. **(D)** The mRNA expression level of *Cg*Astakine. Vertical bars show the mean ± S.D. (*n* = 3); *p < 0.05.

To investigate the function of *Cg*LSD1 in the hemocyte proliferation process, the expression levels of *Cg*CD9, *Cg*SOX11, *Cg*AATase, and *Cg*Astakine in the dsLSD1 group were detected by qRT-PCR after *V. splendidus* injection. The expression level of the agranulocyte-specific marker *Cg*CD9 decreased significantly in the dsLSD1 group, which was about 0.51-fold that of the dsEGFP group (*p* < 0.05) (**Figure 9A**), while the expression level of granulocyte-specific markers *Cg*SOX11 and *Cg*AATase increased significantly in the dsLSD1 group, which were about 3.35-fold and 1.33-fold that of the dsEGFP group, respectively (*p* < 0.05) (**Figures 9B,C**). The expression level of a cytokine-like factor, *Cg*Astakine, also dramatically increased in the dsLSD1 group, which was about 3.52-fold that of the dsEGFP group (*p* < 0.05) (**Figure 9D**).

## 4 Discussion

Histone methylation is recognized as an important modification that is linked to both transcriptional activation and repression (50). An increasing number of reports have documented that histone methyltransferases and demethylases corporately regulate the self-renewal and maintenance of HSCs (50). As an important histone lysine-specific demethylate enzyme, LSD1 was known to play a vital role in both normal and disease state transcriptional programs (31, 51). Although LSD1s have been widely studied in vertebrate species ranging from zebrafish to humans (7, 14, 20, 52), research on LSD1 in invertebrates is very limited, especially in molluscs. In this study, an oyster LSD1 (*Cg*LSD1) was identified in *C. gigas*, and the objective of this study is to explore its molecular features and possible involvement in the hemocyte proliferation during the hematopoiesis process.

LSD1 contains both histone mono-deacetylase and di-demethylase activities (13). The structures of vertebrate LSD1s are known to be very conservative, including an N-terminal SWIRM domain, a C-terminal AO domain, and a central protruding Tower domain (11). In the present study, *Cg*LSD1 from *C. gigas* also contained a SWIRM domain and an AO domain, and the deduced amino acid sequence of *Cg*LSD1 shared high similarity with that of other LSD1 family members, which means that *Cg*LSD1 was found to share high sequence and structure similarity with those from other species. The conserved SWIRM domain in the N-terminal region of *Cg*LSD1 was probably packed against the catalytic domain and presumably contributed to the formation of a substrate-binding groove (53). An AO domain in the C-terminal region of *Cg*LSD1 was proposed to form a large catalytic center where LSD1 was able to mono-demethylate or di-demethylate lysine residues (21). Moreover, a predicted Tower domain consisting of two α-helices was observed in *Cg*LSD1, which might represent a surface for CoREST binding (12). Thus, it is reasonable to suggest that *Cg*LSD1 is similar to LSD1 in other vertebrates in terms of H3K4 demethylase activity (54). To the best of our knowledge, there is another LSD1 homolog protein named LSD2, which lacks the Tower domain and does not have the nucleosome-binding Tower–CoREST architecture (55). In a previous study, the removal of endogenous LSD2 results in an increase in H3K4me2 and a concurrent decrease in H3K9me2 with a consequent downregulation of targeted gene transcription (16). So far, LSD2 has not been reported in invertebrates. The great similarity in protein sequence and structure of *Cg*LSD1 with known LSD1s suggests that *Cg*LSD1 belonged to the LSD1 family in molluscs and might display a similar histone methylation activity in oyster.

The expression patterns of LSD1 in vertebrates are characterized by tissue specificity. The previous reports have demonstrated that LSD1 is expressed ubiquitously and the variability of LSD1 expression may indicate its unique transcriptomes and epigenetic patterns. For example, the protein expression level of LSD1 in both normal and pathologic states of mice was found to be higher in testes than in the somatic tissues (56). The distribution of LSD1 matched the cell- and stage-specific patterns of H3K4 methylation during male germ cell differentiation (57). Furthermore, initiation of LSD1 expression induced the differentiation of some retinal progenitor cells (RPCs), suggesting that LSD1 was highly and uniformly expressed in the eye of mouse and played a critical role in the differentiation of RPCs (58). In this study, the mRNA transcripts of *Cg*LSD1 were widely detected in all the tested tissues with the highest expression level in the mantle and a relatively higher expression level in hemocytes. In molluscs, the mantle is considered to provide protection for the internal soft parts and is related to shell formation, and the edge of the mantle was considered as a vital area for the development of immunocompetence in the mollusc’s larvae (59). The hemocytes of invertebrates are deemed to be the counterpart of vertebrate leukocytes, which are the main immune cells in both humoral and cellular immunity (60). The high expression level of *Cg*LSD1 in mantle and hemocytes suggests its crucial role in oyster immunity and hematopoiesis. LSD1 is also believed to be essential for embryonic development and many physiological processes in vertebrates (28). For instance, as part of the LSD1/CoREST complex, LSD1 is involved in regulating the expression and appropriate timing of vital genes during the early embryonic development of mice, such as brachyury, Hoxb7, and Hoxd8 (61). During the embryonic development of zebrafish, the LSD1 mRNA is expressed during the early cleavage stage and is involved in embryonic patterning (52). *Cg*LSD1 showed lower expression during the embryonic period, while a higher level during the larvae stage, especially in the D-shaped larvae stage with 10.00-fold of that in the zygote stage. According to our previous report, the ring structure around the dorsal region of embryo is considered to be the potential site of hematopoiesis in the trochophore and D-veliger larvae stage (62). The spatiotemporal expression pattern of many hematopoietic transcription factors such as *Cg*GATA2/3, *Cg*SCL, and *Cg*Runx suggests that the sinus structure at the dorsal anterior side of D-veliger should be another potential residue of HSC (59, 63). These findings suggested that *Cg*LSD1 might play a pivotal role during the early development of oysters. The high expression of *Cg*LSD1 in the D-veliger stage implicated its possible involvement in hematopoiesis at the larvae stage. LSD1 has been reported as the nucleus protein, and it is able to decrease the methylation status of lysine residues of histone H3K4 (64). Consistently, *Cg*LSD1 was found to distribute in the nucleus of hemocytes. It was reported that hematopoiesis might participate in immune priming in oysters (35). In the present study, the transcripts of *Cg*LSD1 decreased in hemocytes after *V. splendidus* stimulation. The LSD1 protein was reported to be phosphorylated by a classical protein kinase C (PKCα) after receiving a high dose of LPS, which was crucial for the activation of inflammatory *in vivo* of mice (65). Therefore, the above results suggested that *Cg*LSD1 might play an important role in hematopoiesis during immune response.

In vertebrates, hematopoiesis is a complex process involving many transcription factors which determine the HSCs differentiate into progenitor cells such as the TAL transcription factor (24). As a histone demethylate enzyme, LSD1 is involved in hematopoiesis including cell proliferation and differentiation by regulating specific gene expression. In mammalian cells, multiple factors are associated with LSD1 to regulate its histone demethylase function (14). It was reported that the deletion of LSD1 led to an increased methylation levels of H3K4me1 and H3K4me2 in the stem and progenitor cells of mice, which inhibited the enhancer and promoter activity of HSPC genes and restricted gene expression (66). Similarly, the H3K4me1 and H3K4me2 protein expression level in *Cg*LSD1-RNAi oysters was found to be upregulated significantly in the present study, confirming that *Cg*LSD1 was one of LSD1 family members with conserved histone methylation activity. It has been reported that LSD1 is generally associated with transcription factors to repress the expression of hematopoiesis-related genes during hematopoietic differentiation. For example, the stem cell protein Sal-like Protein 4 (SALL4) plays a crucial part in regulating the growth of hematopoietic progenitor cells and dynamically recruits LSD1 to specific target genes to modulate early hematopoietic precursor proliferation (23). Moreover, the hematopoiesis-specific oncogene T-cell acute leukemia protein 1 (TAL1, also known as SCL) associated with its transcriptional corepressor LSD1 is decreased during erythroid differentiation (24). Several hematopoietic transcription factors including *Cg*GATA2/3, *Cg*SCL, and *Cg*Runx have been reported in oysters; however, the target genes of these transcription factors during the hemocyte differentiation remain unclear. The manner of interaction between LSD1 and hematopoietic transcription factors in oysters needs to be further explored in future studies. In previous research, it was reported that LSD1 was required not only for ESC differentiation [the lack of LSD1 activity in ESCs resulted in failure to fully differentiate (67)] but also for cell differentiation in Merkel cell carcinoma (MCC) (51). The depletion of LSD1 expression increased progenitor numbers by enhancing their proliferative behavior in mice (27). These studies suggested that LSD1 played a vital role in ESC self-renewal and pluripotency in different species. Thus, it is possible for *Cg*LSD1 to participate in maintaining the proliferation and differentiation process in oysters.

Oyster hemocytes are involved in both humoral and cellular immune responses and play vital roles in many biological processes especially in immunological homeostasis (35). In our previous report, the number of hemocytes was found to significantly increase with the dramatic increase in lysosome activity, ROS and NO production, and the relative expression level of immune genes after oysters were stimulated with *V. splendidus* (35, 68). In the present study, EdU was used as an indicator of newborn cells. The percentage of hemocytes with EdU-positive signals in the total hemocytes significantly increased after *Cg*LSD1 was knocked down by RNAi, which was consistent with the knockout of LSD1 in mice (27). Three subtypes of hemocytes have been characterized based on their morphological and physical traits, namely, agranulocytes, semi-granulocytes, and granulocytes (68). Recently, some specific proteins have been identified from the oysters to distinguish three subtypes of hemocytes. For instance, *Cg*AATase and *Cg*SOX11 were characterized as granulocyte-specific markers, and *Cg*CD9 was identified as a specific surface marker for agranulocytes (41, 42). Previous studies suggested that the upregulation of *Cg*SOX11 and *Cg*AATase may represent the differentiation of hemocytes from agranulocytes into granulocytes (69). Agranulocytes are thought to be primitive progenitor cells that differentiate into semi-granulocytes and gradually into granulocytes with different morphological and immunological features (70). In *Cg*LSD1-RNAi oysters, the mRNA transcripts of two potential granulocyte special markers *Cg*AATase and *Cg*SOX11 significantly increased, while the mRNA transcripts of the potential agranulocyte surface marker *Cg*CD9 decreased significantly, partially indicating that the depletion of *Cg*LSD1 in hemocytes induced differentiation from agranulocytes to granulocytes. *Cg*Astakine (a cytokine-like factor homologue to vertebrate) serves as a hematopoietic cytokine involving the proliferation of oyster hemocytes (43). In *Cg*LSD1-RNAi oysters, the mRNA transcripts of *Cg*Astakine significantly increased, indicating the possible involvement of *Cg*LSD1 in the regulation of hemocyte proliferation. These results collectively indicated that *Cg*LSD1 was a hematopoietic lineage-specific modulator and played an essential role as an inhibitory regulator in hemocyte proliferation.

In conclusion, an LSD1 homologue (*Cg*LSD1) was identified in the Pacific oyster with the ability to demethylate H3K4me1 and H3K4me2 in hemocytes. The *Cg*LSD1 expression in hemocytes decreased after stimulation with *V. splendidus*. The percentage of hemocytes with EdU-positive signals significantly increased with an increase in the expression level of granulocyte molecular markers and the hematopoietic cytokine *Cg*Astakine in *Cg*LSD1-RNAi oysters after *V. splendidus* stimulation. *Cg*LSD1 was suggested to participate in the hematopoietic process and might play a pivotal role in the specific gene regulation and hemocyte proliferation of the oyster *C. gigas*.

## Data availability statement

The datasets presented in this study can be found in online repositories. The names of the repository/repositories and accession number(s) can be found in the article/**Supplementary Material**.

## Ethics statement

The animal study was reviewed and approved by Ethics Review Committee of Dalian Ocean University.

## Author contributions

XG designed, performed, and analyzed the experiments; participated in the design of the study; and drafted the manuscript. XQ participated in the design of the study, discussed the results, and reviewed the manuscript. XS participated in the design of the study. SY coordinated the experiment and helped draft the manuscript. LW supervised the manuscript and provided the funding acquisition. LS conceived the study, supervised the manuscript, provided the funding acquisition, and helped draft the manuscript. All authors contributed to the article and approved the submitted version.
