# Supplementary material for: Histone lysine-specific demethylase 1 regulates the proliferation of hemocytes in the oyster Crassostrea gigas
Source: Front Immunol. 2022 Dec 15;13:1088149. doi: 10.3389/fimmu.2022.1088149 (PMC9797820; doi:10.3389/fimmu.2022.1088149)
Supplement: Supplementary file 1 [file DataSheet_1.docx]

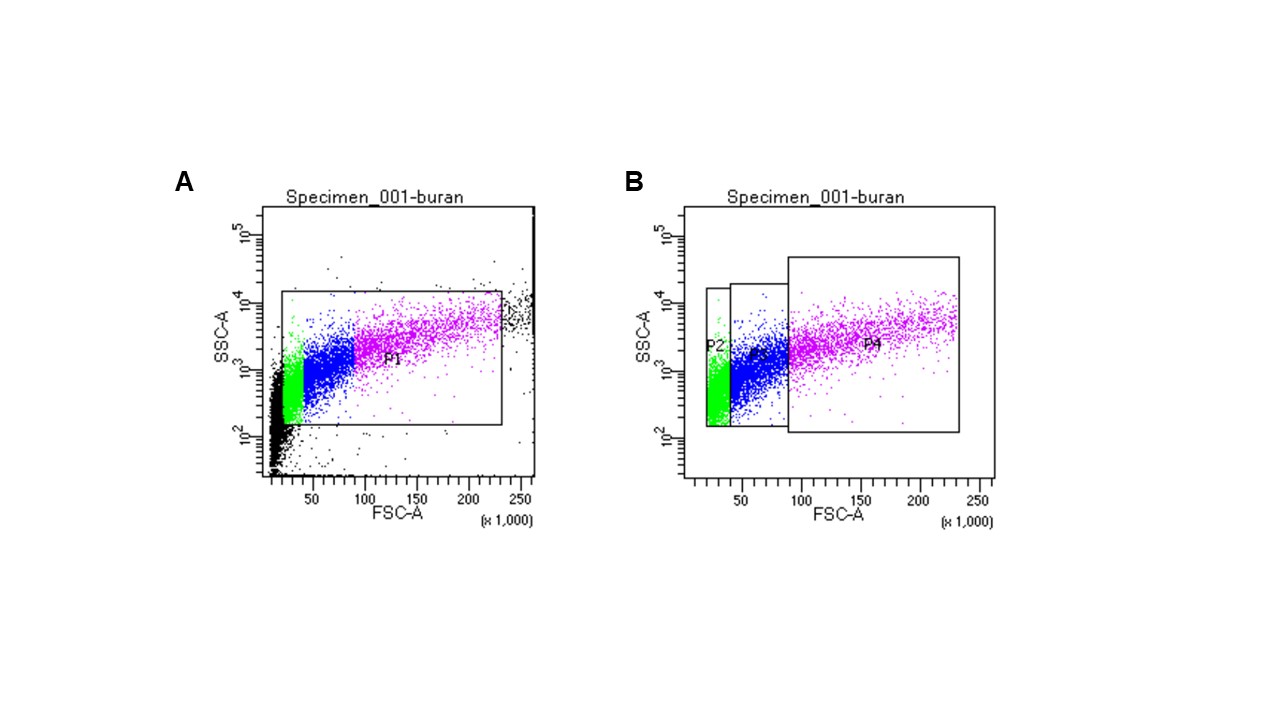


**Fig. S1. The standard of three types of haemocyte gates.**

(A) The total oyster haemocytes were scattered in P1 plot. (B) The agranulocytes were showed in P2 plot which is in the left bottom of the density plots. The semi-granulocytes were showed in P3 plot which is in the middle of the density plots. The granulocytes were showed in P4 plot which is in the right conner of the density plots.
